# Book Reviews

**Published:** 1901-01

**Authors:** 


### Book Reviews.

DEAVER'S SURGICAL ANATOMY. A treatise on human anatomy in its application to the practice of medicine and surgery. By JOHN B. DEAVER, M. D., Surgeon-in-Chief to the German Hospital, Philadelphia. In three royal 8vo volumes of more than 600 pages each, containing about 450 full-page plates, nearly all drawn for this work from original dissections. Volume I.—Upper extremity; back of neck, shoulder and trunk; cranium; scalp; face. Volume II.—Neck; mouth, pharynx, larynx, nose; orbit; eyeball; organ of hearing; brain; female perineum; male perineum. Philadelphia: P. Blakiston's Son & Co, 1012 Walnut St. 1900. [Price, handsome cloth, $21.00; full sheep, $24.00; half green Morocco, marbled edges, $24.00; half Russia, gilt, marbled edges, $27.00 net.]

Twelve years ago Dr. Deaver did the preliminary work on what he intended to be a text-book on surgical anatomy. He was well-equipped for such a task, and the world of surgical literature was in a fair way to receive a most notable addition, one which was sadly needed; for, coming from such a master anatomist as Deaver it could not fail to add to our anatomical knowledge. As he progressed with the work, however, it began to take on new phases and gradually it was broadened in scope.

As an anatomist Deaver ranks among the foremost men in the world, and his skill in this direction dates back to the days of his under-graduate life with that famous class of University of Pennsylvania students, which turned out such men as Price, Dorcum, Roberts, Huidekopper and Anders. It is small wonder, then, that as he got deeply into the work it was changed from a text-book on surgical anatomy to an anatomy, pure and simple, masterly in conception, superb in construction and unapproachable in its marvelous excellence.

This change of plan necessitated considerable enlargement and the work grew to three volumes, two of which have been published. The third will appear in the near future, and then will be completed the greatest work on human anatomy which the world has ever seen.

An idea of the importance attached to this publication may be had when it is known that the plates alone, numbering over 400, cost, according to current statement, in the neighborhood of $40,000. One can hardly wonder at this when a careful study of the illustrations is made. They are the most delicately exquisite, and at the same time most perfect works of their kind which have ever appeared in any work on anatomy. There is not the slightest suspicion of hackneyed work in this connection. In the first place, the illustrations are different from those we have been in the habit of staring at in the perfunctory anatomies of our college days. They are not our old friends. They look new; there is an air of originality about them which sets well, and which is thoroughly in keeping with Deaver's work in the text. And this is all so because every plate was made from drawings of original dissections. They show it and the work speaks eloquently of the years of painstaking conscientious labor which have been devoted to the preparation of the anatomical material from which the drawings were made. Much of this work was performed by Dr. J. Rex Hobensack, who was formerly Dr. Deaver's prosector, and to whom the author gives grateful credit in his preface.

The first volume takes in the anatomy of the upper extremity, back of the neck, shoulder, trunk, cranium, scapula and face. In the second volume we have the neck, mouth, pharynx, larynx, nose, orbit, eyeball, region of the ear, brain, female perineum and male perineum. The third volume, which will soon appear, will contain the abdominal wall, the abdominal cavity, pelvic cavity, chest and the lower extremity.

The chronic pessimist proclaims that nothing is perfect and such an one might, by reason of the distortion of his mental vision find, or imagine he finds, a defect in this work of Deaver; yet his criticism would not be just, because it would not be honest. As a matter of fact, this anatomy is above criticism. It is above honest criticism, because there is nothing about it which honest criticism could conscientiously find to criticise. It is above dishonest, mawkish criticism, because its quiet dignity would damn such a critic before his words were uttered. So let us be content with this and thank Deaver for writing the work and Blakiston for publishing it.

Let us keep Gray handily by, because it is our tried and trusty friend of reference; and Gerrish, because it has clothed the dry, rattling bones of osteology with interest, and Deaver, because it is the best anatomy which has ever been published, and is needed in our more advanced studies and work.

A Reference Handbook of the Medical Sciences. Embracing the entire range of scientific and practical medicine and allied science. By various writers. A new edition, completely revised and rewritten Edited by Albert H. Buck, M. D., New York City. Volume I.—AAC-BLA. Illustrated by numerous chromo-lithographs and 498 half-tone and wood engravings. Nine volumes, imperial 8vo. New York: William Wood & Co. 1900. [Price, muslin, $6.00 per volume; leather, $7.00 per volume; half morocco, $8.00 per volume.]

The republication of this great work has involved an enormous outlay of money as well as the consumption of valuable time. Nevertheless, judging by the appearance of the first volume, it will result in ample compensation to the publishers and all others interested in producing the Reference Handbook.

All the articles have been revised, rewritten or otherwise reconstructed so as to bring each one forward to the present period. One of the most attractive parts of the book consists in the illustrations and these, whether on wood, half-tone plates, or chromo-lithographs, are exquisite specimens of artistic engraving. Take for instance, plate VI., facing page 111, illustrating pigmentation of the skin and of the mucous membrane of the tongue, observed in Addison's disease, and nothing could be more complete in detail or color. Plates X., XI. and XII., illustrating pathogenic bacteria, afford splendid opportunities for study in this field, even to the novitiate.

The text of the articles has been written by men of skill and judgment who possess expert knowledge with reference to the subjects they have prepared for the work: The contributors from Buffalo to this volume are: Herbert M. Hill and Julius Pohlman—names well known to the profession. The letter press deserves mention as being well adapted in clearness and execution to the demands of such a work. We bespeak the interest of the entire profession of medicine in this eminently useful and practical storehouse of knowledge.

---

TWENTIETH CENTURY PRACTICE. An International Encyclopedia of Modern Medical Science. By leading authorities of Europe and America. Edited by THOMAS L. STEDMAN, M. D., New York City. In twenty volumes. Volume XX. "Tuberculosis, Yellow Fever and Miscellaneous. General Index." New York: William Wood & Company. 1900.

With the beginning of the twentieth century we fittingly present a notice of the last volume of the Twentieth Century Practice. It bespeaks great industry and enterprise that the editor and publishers have been able to complete a work of this magnitude in importance within the period of time which they allotted themselves at the outset. The most important subject considered in this volume is tuberculosis. August Jerome Lartigau deals with its bacteriology, pathology and etiology; Henry W. Berg considers its symptomatology; S. A. Knopf discourses upon its diagnosis, prognosis, prophylaxis and treatment, and John T. Bowen considers tuberculosis of the skin. These four essays together, form a most elaborate and discursive treatise upon a disease that is now known to be not only infectious but preventable.

Yellow fever is the next topic which is presented in this volume and it is written by Wolfred Nelson. Since tropical islands have become a part of our domain this destructive malady has come to be considered in the light of prevention. Heretofore our only remedy has been through rigid quarantine against infected districts. Now, by applying the scientific principles of sanitation at the sources of the disease we may expect to stamp it out with reasonable certainty.

The miscellaneous subjects considered in this volume are: Poisoning with snake venom, by Thomas R. Brown; Mushroom poisoning, by Beaumont Small; Diseases of the uvula, soft palate and faucial pillars, by James E. Newcomb, and neural and mental defects in childhood, by Francis Warner. The general index then follows which consists of very nearly 300 pages. This is a most comprehensive index which enables the searcher to find any title at short notice.

In concluding this notice we feel it appropriate to thank the editor and the publishers for this comprehensive and learned work upon medicine, that in and of itself constitutes a library of no mean proportions, and contains some of the best literature extant on the subjects of which it treats.

THE STUDENT'S MEDICAL DICTIONARY. Including all the words and phrases generally used in medicine, with their proper pronunciation and definitions. Based on recent medical literature. By GEORGE M. GOULD, A. M , M D , Author of "An Illustrated Dictionary of Medicine, Biology and Allied Sciences," etc. Large 12mo. Eleventh edition, enlarged. Illustrated. Philadelphia: P. Blakiston's Son & Co. 1900. [Price, $2.50.]

This edition of this popular dictionary has been increased in size to the extent of over a hundred pages more than its predecessor. The author is an experienced maker of word books and is in touch with the needs of students in the premises. It is not difficult to discover even through a cursory examination that this work is especially adapted to the wants of medical students. Its size is admirable, it furnishes definition and pronunciation of all the terms that are in general use, while it is excellently illustrated and its tables are beyond criticism.

We regard it as one of the most convenient, compact and at the same time scientifically accurate of all the student's medical dictionaries afloat.

---

STRINGTOWN ON THE PIKE. A Tale of Northernmost Kentucky. By JOHN URI LLOYD, Author of "Etidorpha," etc. With illustrations. New York: Dodd, Mead & Co. 1900.

This may be termed a study of Kentucky character, the scenes of which are laid in a northern Kentucky hamlet, but ten miles away from Cincinnati. This region is in strong contrast to the blue grass section where are laid the scenes of James Lane Allen's stories.

The characters are such as belong to the region named, and barring the sometimes almost impossible dialect, it makes pleasant reading and a restful diversion for the medical man. Some of the chapters, too, such as 39 and 40, 55 to 63, and this final chapter possess professional interest. The story has been running through the *Bookman* since March, 1900, where it has attracted widespread attention on account of its literary merit as well as its attractiveness as a story. Its word pictures are strong, its dialogue amusing and its moral tone is high. The publishers have dressed it in attractive type. We commend it to our readers.

THE PHYSICIAN'S VISITING LIST (Lindsay and Blakiston's) FOR 1901. Fifteenth year of its publication. Philadelphia: P. Blakiston's Son & Co., 1012 Walnut street.

Few publications of any sort have reached their fiftieth anniversary, yet this popular visiting list enjoys that distinction. Moreover, it well merits any claim to honor on that account, for it is one of the most compact, convenient and best visiting lists published. It contains all that is needed for reference by the busy, practical, everyday doctor, in addition to the regular pages devoted to records of patients, arranged in weekly blocks. Those who have used it before will be sure to need it now and many many others will flock to its standard.

